# Health Consequences of COVID-19 Pandemic in Older Adults with Musculoskeletal Conditions: A Cross-Sectional Path Analysis Model

**DOI:** 10.3390/geriatrics10060139

**Published:** 2025-10-27

**Authors:** Suparb Areeue, Inthira Roopsawang, Rick Yiu Cho Kwan, Ladda Thiamwong

**Affiliations:** 1 Ramathibodi School of Nursing, Faculty of Medicine Ramathibodi Hospital, Mahidol University, Bangkok 10400, Thailand; suparb.are@mahidol.ac.th; 2 School of Nursing, Tung Wah College, Hong Kong; rickkwan@twc.edu.hk; 3 College of Nursing, University of Central Florida, Orlando, FL 32827, USA; ladda.thiamwong@ucf.edu

**Keywords:** depressive symptoms, fear of falls, health consequences, older adults, physical activity, social frailty

## Abstract

**Background/Objective**: The sequelae of COVID-19 on geriatric health is profound, yet its consequences on mental well-being remain insufficiently elucidated, particularly in older adults with musculoskeletal conditions. This study aimed to explore the interrelationships and magnitude of the effects of fear of COVID-19, fear of falls, physical activity, and social frailty on depressive symptoms in this population. **Methods**: Purposive sampling was applied to recruit 292 older adults with musculoskeletal conditions. Data were collected through structured interviews (face-to-face and telephone) using standard questionnaires. Path analysis with Satorra–Bentler correction examined the relationships in the proposed model of depressive symptoms. The model fit indices were evaluated using the chi-square (χ^2^); the goodness-of-fit test was assessed with standard criteria of the comparative fit index (CFI ≥ 0.95), the Tucker–Lewis index (TLI ≥ 0.95), the root mean squared error of approximation (RMSEA < 0.08), and the standardized root mean square residual (SRMR < 0.05). **Results**: Mean participant age was 70.30 ± 6.56 years, with 74.3% female. The path analysis model demonstrated an excellent fit indicating χ^2^ = 0.007 (*p* = 0.933), CFI and TLI = 1.000, RMSEA = 0.000, SRMR = 0.001. Fear of COVID-19 negatively indirectly impacted depressive symptoms (β = −0.07, *p* = 0.017), while physical activity had a positive direct effect (β = 0.16, *p* = 0.004). Fear of COVID-19 directly influenced social frailty (β = 0.18, *p* = 0.003) but had a negative direct impact on physical activity (β = −0.37, *p* = 0.000). However, fear of falling did not show a significant relationship with the other study variables. **Conclusions**: Depressive symptoms entail physical and psychosocial consequences. Physical activity has a positive effect on depressive symptoms. Fear of COVID-19 increases social frailty, while increasing physical activity reduces this fear. Future research should evaluate longitudinal effects and investigate evidence-based public health interventions or tailored cognitive–behavioral interventions to reduce pandemic-related fear and prevent mental health sequelae.

## 1. Introduction

The sequelae of depressive symptoms in older adults have a substantial influence on their overall health and quality of life on a global scale. Once the COVID-19 pandemic emerged in 2019, global priorities shifted toward health consequences, as the virus may potentially cause unforeseen impacts on older adults, particularly mental health problems [1,2,3]. As noted, the intractable disease has raised uncertain circumstances related to COVID-19; empirical studies have highlighted a possible connection to an increasing prevalence of depressive symptoms in older adults [2,4]. Unpredictable circumstances—the rapid spread of variant mutations, uncertainties regarding vaccination, and various implemented control or restriction policies—have led to several health issues, including physical, psychological, and social consequences [4,5,6]. Moreover, the ongoing COVID-19 pandemic has profoundly impacted not only individuals’ daily activities [5,7] but also their mental health [1,2]. Among older adults, various mental instabilities such as fear of COVID-19 infection, stress, and anxiety disorder have been observed, while depressive symptoms are a significant concern in this population [1,6]. Additionally, multiple factors may contribute to heightened feelings of uncertainty or fear, resulting in more complex psychological problems or depressive symptoms among older adults. The increasing fear of COVID-19, physical and social restrictions, disrupted daily activity routines, and the burden of chronic illness, the higher risk of developing depressive symptoms [2,5,8]. As the nature of aging is a multifaceted phenomenon, depressive symptoms seem to be more complex in late-life older adults [2,6], potentially intensifying the burden of care in this population. Although numerous studies exist on this topic, gaps in care persist. Few studies have focused on age-related changes that influence depressive symptoms in older adults with musculoskeletal conditions (MSK) [6,9]. Hence, understanding the factors influencing depressive symptoms is essential to prevent or mitigate its consequences. Moreover, providing effective management of depressive symptoms—preventing consequences or promoting health through alteration of risk factors—for older adults is more challenging for healthcare personnel.

All ages have faced the conundrum of COVID-19; however, older people with multiple comorbidities seem to suffer more from this situation. Due to aging processes, older adults predominantly present with a geriatric syndrome—frailty—that intensifies the age-related changes across multiple health systems [10]. Current evidence revealed that frailty is associated with MSK; conjointly, MSK is an indicator predicting frailty, poor health outcomes, physical limitation, and disability [11]. Accordingly, the prevalence of frailty in older adults with MSK and its impact might simultaneously increase because of uncertain circumstances, restrictions, or preventive measures against COVID-19 infection. Although the effects of frailty have primarily been investigated in terms of physical and psychological dimensions, other dimensions—social and environmental—are of concern. Recently, social frailty, a new perspective, has emerged, highlighting the social dimension of individuals who are frail [12]. The social frailty perspective considers the interaction between intrinsic factors (physical frailty, comorbidity, and genetics) and extrinsic factors (social, physical, environmental, and economic conditions) to justify social frailty determinants [8,13]. Recent evidence has revealed that social frailty predicts adverse outcomes, including cognitive impairment, muscle weakness, mobility impairment, physical frailty, and increased mortality [8,13]. Undoubtedly, social frailty may perpetuate a vicious cycle of the burden of care, resulting in an increased demand for long-term care and reduced quality of life, particularly in older adults with MSK. Accordingly, the detriment of social distancing may be a potential silent stressor. The social distancing policy may prevent disease-spreading, yet it is more likely to be the other significant stressor affecting older adults at risk of social frailty, resulting in more severe depressive symptoms [7,14].

During restricted social interaction, the impact of social frailty on physical and mental function and quality of life has become more of a concern for healthcare providers. Current evidence has demonstrated the association between social frailty and fear of falling on quality of life; fear of falling has direct and indirect effects on physical and mental aspects [15,16]. Accordingly, older adults who fear falling mainly experience limitations in their daily physical activity (PA), resulting in a decreased overall quality of life [17]. Remarkably, in older adults, the COVID-19 pandemic has caused increased fear, perceived risks for loved ones, and health anxiety [18]. The fear of COVID-19 has been associated with social isolation, fear of movement, and decreased physical activity levels among older individuals, potentially contributing to an increased risk of falls [19]; physical distancing and fear of COVID-19 may also have reduced routine healthcare visits. Evidence also suggests that the pandemic has led to a decrease in physical activity and other deconditioning among older adults, which has been associated with increased depressive symptoms and decreased quality of life [20]. The impact of the COVID-19 pandemic may have more profound consequences both in the short- and long-term on the health of older people.

Controlling disease spreading without treatment guidelines is rudimentary, yet more concerns are to come. Older adults with MSK who have physical limitations or physical frailty might suffer more from social frailty; conjointly, MSK directly impacts physical performance, which influences poor physical activity and increases the risk of disability [21]. Additionally, social frailty may intensify functional limitation, physical frailty, or increase the severity of disability [13]. Unquestionably, older adults with MSK conditions are more likely to suffer from both physical and mental problems, resulting in diminished quality of life. There is also a dearth of knowledge about the extent to which factors—fear of COVID, fear of falling, physical activity, and social frailty—affect depressive symptoms in this population. Understanding the associations between these important health variables would direct the development of assessment methods to identify older adults at risk of poor health outcomes and develop digital health interventions to promote their health during the pandemic. The study aimed, therefore, to investigate the complex relationships—direct and indirect—of fear of COVID-19, fear of falling, physical activity, and social frailty on depressive symptoms in older adults with MSK.

## 2. Materials and Methods

### 2.1. Study Design, Setting, and Participants

This cross-sectional descriptive study followed the STROBE guideline, focusing on the main study variables, including fear of COVID-19, fear of falling, physical activity, social frailty, and depressive symptoms. The prospective participants were older adults with musculoskeletal conditions who visited the orthopedic outpatient department of a tertiary care university hospital in Bangkok, Thailand, in June–October 2021. The prospective participants were required to meet the following criteria: aged 60 years or older, currently presenting with musculoskeletal conditions, no cognitive impairment assessed by the Six-Item Cognitive Impairment Test–Thai version (6-CIT) (0–7 points) [22], and no hearing, seeing, or speaking impairment that affects face-to-face or telephone interviews. Those who were dependent or had bedridden conditions were excluded from the study.

### 2.2. Sample Size

The Cochran formula N = (Z^2 *p* (1 − *p*))/d^2 was applied for sample size calculation. The sample size was considered 95% of the significance level, the power of analysis (power = 0.95), and Z = 1.96. The calculated sample size was approximately 146–292. In this study, 292 cases were recruited for final analysis.

### 2.3. Data Collection

After receiving the Institutional Review Board (IRB) approval, the recruitment processes were employed at the orthopedic outpatient department; the eligible participants, based on the purposive criteria, were informed and invited to participate in this study. The prospective participants were screened for cognitive impairment using the 6-CIT. Participants who met the inclusion criteria were asked to sign informed consent forms before trained research assistants (RAs) collected data. For those who did not meet the inclusion criteria, health education related to their request was given. The interviews were conducted using either face-to-face or telephone interviews and Electronic Medical Record (EMR) reviews of older adults with musculoskeletal conditions who visited the hospital. Older adults have the right to refuse participation without any effect on hospital services; those who voluntarily agreed were called for data collection.

### 2.4. Ethical Consideration

The study was conducted in accordance with the Declaration of Helsinki and received approval from the Institutional Review Boards (IRBs) of the Faculty of Medicine Ramathibodi Hospital, Mahidol University (COA. MURA2021/891). Informed consent was obtained from all participants involved in the study. Data collection was performed after participants received a clear explanation of the study’s aims, methods, risks, and benefits, and they provided verbal or written informed consent. Participants were also informed of their right to choose whether or not to participate, without any impact on the health services they would receive. All study information was kept confidential, reported in groups, and presented solely for educational purposes.

### 2.5. Measures

#### 2.5.1. Demographic and Health Information

The Demographic and Health Information was designed to extract information related to participants’ characteristics (age, gender, marital status, education level, medical payment, and residence status) and health information (pain, health-related conditions, comorbidity, and number of medications).

#### 2.5.2. The Six-Item Cognitive Impairment Test (6-CIT)

The 6-CIT, a brief, self-reported, and less time-consuming (completed in 2 min), is a feasible cognitive screening in hospital and community settings for older adults [23]. The 6-CIT was translated into many languages, including Thai [22]. The 6-CIT consisted of 6 questions: a logical memory test, two attention tests, and three orientation questions. The total score of 6-CIT ranged from 0–28; the higher scores indicate the increased severity of cognitive impairment, while a score of 7 or lower indicates cognitive abilities are intact. The 6-CIT showed excellent reliability and validity in clinical predicting cognitive impairment, delirium, and dementia [24]. The Thai version of 6-CIT also demonstrated a good prediction of cognitive impairment in Thai older adults [22].

#### 2.5.3. Fear of COVID-19 Scale (FCV-19)

The FCV-19, developed by Ahorsu and colleagues [25], was used to evaluate the consequences of COVID-19, which have led to fear, worries, and anxiety. The FCV-19S consists of seven items that inquire about an individual’s perception of the fear of COVID-19. A 5-point Likert scale, ranging from “strongly disagree” (1 point) to “strongly agree” (5 points), is utilized to determine the level of agreement with the statements regarding COVID-19. The total score of FCV-19S ranges from 7 to 35; the higher scores indicate a higher fear of COVID-19. The FCV-19S is a reliable instrument with a demonstrated internal consistency of 0.82. Furthermore, it has shown strong concurrent validity through significant correlations with standard measurements—the Hospital Anxiety and Depression Scale (HAD) and the Perceived Vulnerability to Disease Scale (PVDS) (*p* < 0.001) [25].

#### 2.5.4. The Fall Efficacy Scale-International (FES-I)

The FES-I was used to measure the fear of falling [26]. The shortened version of FES-I comprises seven items measuring how much the subjects are concerned about their falls when participating in seven activities of daily living. Each item could be rated on a 4-point scale (from 1 = “not at all concerned” to 4 = “very concerned”) with total scores from 7 to 28. A higher score indicates a higher level of fear of falling. FES-I was validated to have a strong correlation with the extended version (r = 0.97) and excellent Test–Retest reliability (Intraclass correlation coefficient (ICC) = 0.83) and internal consistency (Cronbach’s alpha = 0.92) [26].

#### 2.5.5. The Rapid Assessment of Physical Activity (RAPA)

The RAPA was employed to assess physical activity [27]. The RAPA comprises nine dichotomous items, with 7 items in part 1 (1 = yes, 0 = no) and 2 items in part 2 related to strength and flexibility (if the answer is yes to both items, a score of 3 will be given). Part 1 asks about levels of physical activity, while Part 2 focuses on strength and flexibility exercises. For this study, only part 1 was used. The RAPA classifies physical activity levels into seven levels by intensity (from 1 = sedentary to 7 = regular active). The cut-off point of the RAPA is 6, which means any point below six is considered a suboptimal level of physical activity. It has been validated in older adults and shows acceptable validity with a strong correlation with the Community Health Activities Model Program for Seniors (CHAMPS) (r = 0.54) [27].

#### 2.5.6. The 5-Item Social Frailty Questionnaire

This measure was used to assess social frailty. It is a self-report measurement developed by Makizako and colleagues [28]. There were five questions with binary answers (yes/no). The answer “yes” to questions 1 and 4 and “no” to questions 2, 3, and 5 were considered negative responses. The total scores based on negative responses were counted and then classified into the different health statuses: frailty (2–5), prefrail (1), and robust (0). The 5-item social frailty has been employed to measure social frailty in older populations [13].

#### 2.5.7. The Patient Health Questionnaire (PHQ-9)

The PHQ-9 was used to assess depressive symptoms [29]. The PHQ-9 is a self-administered screening tool designed to evaluate depressive symptoms. PHQ-9 comprises nine items that inquire about the frequency of mental discomfort experienced in the preceding two weeks. Scoring of each item is based on a 0–3 scale (0 = not at all; 1 = several days; 2 = more than a week; 3 = nearly every day). The total score of the PHQ-9 ranges from 0–27 and is used to classify the severity of depressive symptoms as mild (scores of 5–9), moderate (scores of 10–14), moderately severe (scores of 15–19), and severe (scores of 20). The PHQ-9 has been evaluated in various specific populations with different cultural and linguistic contexts, exhibiting high internal reliability (Cronbach’s α = 0.89) and Test–Retest reliability (ICC = 0.89, 95% CI 0.85–0.91) [30].

All measurements used in this study were authorized and permitted.

### 2.6. Statistical Analysis

Data analysis was conducted using the licensed STATA Version 18; details were as follows:

1. Descriptive statistics were computed to report the percentage, mean, and standard deviation of personal data and the average scores of all variables in the study.
2. A path analysis with Satorra–Bentler correction was employed to investigate the relationships and magnitudes of the effects of fear of COVID-19, fear of falling, physical activity, and social frailty on depressive symptoms. Statistical significance was set at *p* < 0.05. The model fit indexes were evaluated using the chi-square (χ^2^), the goodness-of-fit test, the comparative fit index (CFI), the Tucker–Lewis index (TLI), the root mean squared error of approximation (RMSEA), and the standardized root mean square residual (SRMR). The following criteria were required to demonstrate an acceptable fit: the CFI ≥ 0.95, TLI ≥ 0.95, RMSEA < 0.08, and SRMR < 0.05.

## 3. Results

### 3.1. Demographic Characteristics

A total of 292 older people with MSK conditions participated; of these, the majority were female (74.3%) with a mean age of 70.30 years (standard deviation (SD) = ±6.56). Most participants were married (51%), graduated from primary school (38.3%), received government reimbursement for medical expenses (65.4%), and lived with their families (73.6%). Regarding health status, more than half suffered from musculoskeletal conditions related to the knee (36.6%) and spine (29.8%). Additionally, a considerable proportion of the participants reported bodily pain (71.2%), had comorbidities (79.5%), predominantly hypertension and dyslipidemia, and used at least one medication (68.2%) (as shown in Table 1).

### 3.2. The Association Among Study Variables

The findings of the descriptive analysis of the study variables are presented in Table 2. According to the correlation matrix of the study variables (Table 3), Spearman’s rho correlation coefficient (r_s_) demonstrated a significant association with depressive symptoms, indicating a negative direction of fear of COVID-19 (rs = −0.158, *p* < 0.05). In contrast, a positive direction of physical activity (rs = 0.220, *p* < 0.05) was identified. Non- statistically significant correlations on depressive symptoms were discovered: a positive association with fear of falling (rs = 0.071, *p* = 0.23) but approached a negative association with social frailty (r_s_ = −0.099, *p* = 0.09).

**Figure 1:**
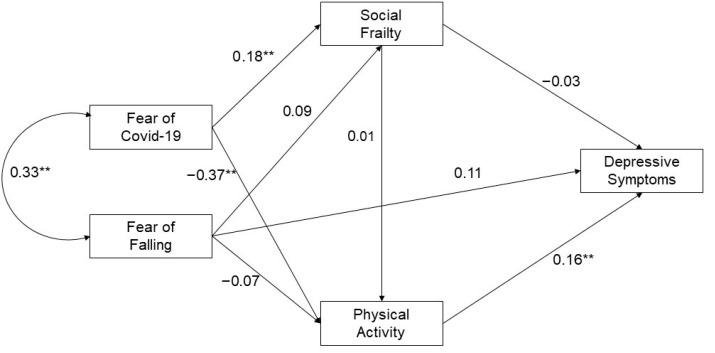
Path model of the association among health consequent factors in older people with MSK conditions during COVID-19. Overall model fit, chi-square (χ^2^) = 0.007, *p* = 0.933, comparative fit index = 1.000, Tucker–Lewis index = 1.000, root mean squared error of approximation = 0.000, standardized root mean squared residual = 0.001. Significant at ** *p* < 0.01.

The path analysis model demonstrated an excellent fit for explaining the nexus link among selected factors on depressive symptoms in older adults with MSK conditions, as depicted in Figure 1. As shown in Table 4 and Figure 1, the complex effects on depressive symptoms were identified. Specifically, in physical activity, both positive and negative effects were evident in its direct and indirect pathways; notably, a significant negative direct effect was identified in fear of COVID-19 (β = −0.37, *p* = 0.000), while others revealed non-significant associations. Regarding social frailty pathways, only the direct effect of fear of COVID-19 was observed (β = 0.18, *p* = 0.003). Remarkably, for significant effects on depressive symptoms, fear of COVID-19 had a significant negative indirect effect (β = −0.07, *p* = 0.017), whereas physical activity had a significant positive direct effect (β = 0.16, *p* = 0.004). However, fear of falling and social frailty had non-significant direct effects on depressive symptoms.

## 4. Discussion

The findings of this study, which may be the first in initial investigations of health consequences in older adults with MSK conditions, demonstrated a strong model fit in explaining the link underpinning health consequences in this population. Our findings highlighted the complex interplay of health consequences—physical, mental, and social dimensions—during the pandemic. The pervasiveness of depressive symptoms was observed, indicating mental health challenges. The complex nexus of multi-dimensional impacts was also well-established, revealing that the fear of COVID-19 is not only associated with the fear of falling but also contributes to less physical activity and heightened severity of social frailty, resulting in worsening depressive symptoms.

A notable finding in our study was a substantial indirect pathway linking fear of COVID-19 to depressive symptoms, which is mediated by the direct effect of physical activity. Additionally, increased fear of COVID-19 was found to be correlated with a heightened fear of falling in this population. Based on the study’s findings, these interrelated effects of fear of COVID-19 align with prior evidence suggesting a profound psychological impact on health. Several possible mechanisms have been proposed to elucidate this phenomenon [31,32]. Growing evidence has emphasized that overwhelming context—implementation of preventive measures and strict policy implementation during unpredictable situations—may play a role in intensifying fear related to COVID-19, subsequently leading to physical and social limitations that disrupt daily activity routines. These disruptions can increase the risk of reduced physical activity, limited social interaction, or social frailty, resulting in increased depressive symptoms [5,7,8]. Regarding physical aspects, evidence revealed that a heightened fear of COVID-19 is associated with diminished physical activity levels in older adults; this decline in physical activity potentially contributes to an increased risk of falls [19]. Undoubtedly, in older adults with MSK conditions, the aging process, on top of MSK problems, may hasten the decline of musculoskeletal function, leading to decreased physical activity [31]. Furthermore, the disuse syndrome in older adults with MSK conditions can result in accelerated muscle loss and functional impairment, which may increase physical limitations and impaired gait balance, ultimately increasing risks of falls [31,32]. As mentioned earlier, the reduction in physical activity stemming from COVID fear [2,19], coupled with an age-related physical limitation [33], may exacerbate functional decline, leading to increased care dependency or disability. Consequently, these interconnected issues eventually elevate the risk of developing severe depressive symptoms in this population. While the advantages of promoting physical exercise to prevent depression symptoms in older persons are widely supported by evidence, it is challenging to facilitate physical activity during the appropriately controlled pandemic; however, there is no solution that is applicable in all circumstances. Further work is needed to investigate specific populations, particularly older people with MSK conditions.

Regarding the social aspects, contrary to expectations, our findings demonstrated that social frailty has a non-significant negative direct impact on depressive symptoms. Although the notable significant association between social frailty and depressive symptoms has been reported [8,13,34,35], our finding was somewhat different from previous reports regarding the link between social frailty and depressive symptoms in older adults. These results should be interpreted cautiously, as latent factors or hidden dimensions that may influence social frailty have not been fully identified during the pandemic. Remarkably, the new insight that our findings revealed here is that the relationship between social frailty and depressive symptoms in older adults with MSK conditions appears to be more nuanced than previously thought. Recent evidence underlined that the link between social support and depressive symptoms is primarily indirect, particularly for older adults who are frail [36]. Therefore, it could be inferred that the interrelated link between social frailty and depressive symptoms may not always manifest as a direct association. Instead, this link may be influenced by other mediating factors—social support, physical capacity, and physical activity in daily living. In older adults with MSK conditions, reasons for avoiding social participation might be due to acknowledging the actual functional decline of physical limitation because of suffering from MSK conditions [31,37]. During the pandemic, such social distancing and preventive measure restrictions temporarily marked this limitation for COVID control. Moreover, Thailand’s simultaneous deployment of vaccination campaigns with social restrictions may be considered as government social support, potentially contributing to mitigating pandemic-related stress. Hence, even if the risk of social frailty increases, limited social participation or interaction may enhance safety in disease prevention, resulting in lessened depressive symptom severity for older adults with MSK conditions. Conversely, lessened social frailty severity with increasing social participation can potentially lead to the development of depressive symptoms during the pandemic situation. Increased social participation with heightened physical activities may worsen pain and physical discomfort, which could lead to feelings of frustration, uncertainty, and dependence, contributing to heightened stress levels and, consequently, developing depressive symptoms more severely [38]. This specific phenomenon in older adults with MSK conditions has been divulged by our findings, revealing that increasing physical activity has a positive direct effect on increasing depressive symptoms.

In addition to the earlier mediating factor altering the connection between social frailty and depressive symptoms, recent studies have suggested that personal characteristics and cultural aspects, such as individual resilience, strong informal social support, cultural influences, and engagement in meaningful activities, may enhance or negate the link between social participation and depressive symptoms [36,39,40]. Notably, in traditional Thai culture, the support from social and family networks and key caregivers may be vital in mitigating stress, even in situations with limitations in social participation. Moreover, prior evidence has underlined that lessened exposure to pandemic-related stressors or anxiety-inducing information may potentially reduce depressive symptoms, particularly during the pandemic situation [41]. However, recent work has emphasized that reduced social activities were not associated with depressive disorder, while lessened family gatherings were double the risk of developing depressive disorders in older adults during the COVID-19 pandemic [42]. Moreover, the pandemic-related restriction policies in Thailand led to increased family living together; this situation potentially enables younger family members to assist their older generation with daily tasks such as grocery shopping, housekeeping, and meal preparation. As a result, Thai older adults with MSK conditions may have engaged in these activities with family members, possibly contributing to a reduction in fear of falling. Hence, our findings suggest that initiating effective physical activity interventions for older adults requires careful consideration of varying levels of fear, physical activity, and diverse sociocultural backgrounds.

### Strengths and Limitations

As mentioned, during the pandemic, older adults may experience depressive symptoms due to the nexus of various factors—increased fear of COVID-19, heightened fear of falling, enhanced active physical activity, and lessened social frailty. When it comes to holistic care, caring for physical, social, and mental dimensions is essential for delivering comprehensive care. However, it is crucial to recognize that providing professional care that serves personalized care needs could yield stronger benefits in improving care quality and preventing the spread of the COVID-19 pandemic. In this present study, while the nexus pathways may be well-established, the findings should be interpreted with caution. Due to the study design, the robust association observed does not reflect causal relationships, and confounding factors were not accounted for in the model. Hence, focusing more on examining a multifaceted and interdependent connection among psychological, physical, and social aspects is recommended for older adults with MSK conditions. Additional analyses will be required to establish holistic care preparedness for potential future pandemics.

## 5. Conclusions

The study highlights the meaningful impact of geriatric health consequences on older adults with MSK conditions, specifically in relation to the complex factors that influence depressive symptoms. An increasing fear of COVID-19 has a considerable impact on increased depressive symptoms through physical activity. It is noteworthy that increased physical activity may paradoxically lead to an exacerbation of depressive symptoms in this population. The findings of this study have raised points of concern about the complex relationship between depressive symptoms and latent factors that may have a substantial impact on the psychological well-being of older adults with MSK conditions, particularly during challenging periods such as the COVID-19 pandemic. Therefore, a comprehensive understanding of these variables and depressive symptoms may facilitate tailored intervention or the development of responsive policy approaches for older adults with MSK conditions by considering their unique needs and available resources.

## Figures and Tables

**Table 1:** Characteristics of study participants.

Variables	n (%)	Variables	n (%)
**Age** (**years**)		**Residence status**	
60–69	147 (50.3)	With family	215 (73.6)
70–79	119 (40.8)	With spouse	51 (17.5)
≥80	26 (8.9)	Living alone	26 (8.9)
(Mean ± SD = 70.30 ± 6.56)			
		**Health**-**related conditions**	
**Gender**		Knee	107 (36.6)
Female	217 (74.3)	Spine	87 (29.8)
Male	75 (25.7)	Ankle	26 (8.9)
**Marital status**		Hip	9 (3.1)
Married	149 (51.0)	Others (i.e., bone tumors)	63 (21.6)
Widowed/divorced	114 (39.1)	**Comorbidities**	
Single	29 (9.9)	None	60 (20.5)
**Education**		Yes	232 (79.5)
Informal	4 (1.4)	Hypertension	125 (42.8)
Primary	112 (38.3)	Dyslipidemia	113 (38.7)
Secondary	23 (7.9)	Diabetes mellitus	47 (16.1)
Associated degree	18 (6.2)	Congestive heart failure	15 (5.1)
Bachelor’s degree	105 (35.9)	Carcinomas	9 (3.1)
≥Master’s degree	30 (10.3)	Others (i.e., COPD, CVA)	87 (29.8)
**Medical service**		**Bodily pain**	
Government reimbursement	191 (65.4)	Yes	208 (71.2)
Self-paid	46 (15.8)	None	84 (28.8)
Health insurance	38 (13.0)	**Medication usage** (**numbers**)	
Social security	10 (3.4)	None	93 (31.8)
Universal health coverage	7 (2.4)	1–3	122 (41.8)
		≥4	77 (26.4)

Abbreviations: COPD = Chronic obstructive pulmonary disease; CVA = Cerebrovascular accident; SD = Standard deviation.

**Table 2:** Descriptive analysis of the study variables.

Variables	Possible Scores	Actual Scores	Mean ± SD
Fear of COVID-19	7–35	7–30	13.47 ± 4.66
Fear of falling	7–28	7–21	10.17 ± 3.16
Physical activity	1–7	1–7	4.27 ± 1.77
Social frailty	0–5	0–4	1.99 ± 0.56
Depressive symptoms	0–27	0–11	1.08 ± 1.86

**Table 3:** The correlation matrix of the study variables (N = 292).

Variables ^a^	1	2	3	4	5
1. Fear of COVID-19	1.000				
2. Fear of falling	0.364 **	1.000			
3. Physical activity	−0.445 **	−0.190 **	1.000		
4. Social frailty	0.219 **	0.200 *	−0.093	1.000	
5. Depressive symptoms	−0.158 *	0.071	0.220 *	−0.099	1.000

^a^ Spearman correlation analysis; significant at *p* < 0.001 ** and *p* < 0.05 *.

**Table 4:** Standardized direct and indirect effects of exogenous and endogenous variables (N =292).

Variables	Physical Activity		Social Frailty		Depressive Symptoms	
	DE ^a^	IE ^b^	DE ^a^	IE ^b^	DE ^a^	IE ^b^
Fear of COVID-19	−0.37 **	0.001	0.18 **	-	-	−0.07 *
Fear of falling	−0.07	0.001	0.09	-	0.11	−0.01
Physical activity	-	-	-	-	0.16 **	-
Social frailty	0.01	-	0.01	-	−0.03	0.001

^a^ Direct effect (DE); ^b^ Indirect effect (IE); significant at *p* < 0.001 ** and *p* < 0.05 *.

## Data Availability

Due to the nature of this research, participants of this study did not agree that their data should be shared publicly, so supporting data is not available.

## Abbreviations

The following abbreviations are used in this manuscript:

CFI	Comparative Fit Index
COVID-19	Coronavirus Disease-19
EMR	Electronic Medical Record
MSK	Musculoskeletal Conditions
PA	Physical Activity
RMSEA	Root Mean Squared Error of Approximation
SRMR	Standardized Root Mean Square Residual
TLI	Tucker–Lewis Index
